# Facile Fabrication of Hierarchical Multimodal Nanoporous Gold (hm-NPG) via a Polysaccharide Polymer Template Method

**DOI:** 10.3390/nano16150916

**Published:** 2026-07-25

**Authors:** Taiwo Musa Adeniji, Palak Sondhi, Cailey Shanks, Jagan Rajamoni, Keith J. Stine

**Affiliations:** Department of Chemistry and Biochemistry, University of Missouri–St. Louis, Saint Louis, MO 63121, USA; tmaqmy@umsl.edu (T.M.A.); palaksondhi17@gmail.com (P.S.); cshanks222@gmail.com (C.S.); jrbrm@umsl.edu (J.R.)

**Keywords:** nanoporous gold, template, dextran, multimodal, dealloying

## Abstract

Dealloyed nanoporous metals have a unique bicontinuous solid/void structure that provides a sizable surface area and outstanding electrical conductivity, making them attractive candidates for use in a range of applications. But for many of these applications, the utilization of an engineered hierarchical porous network topology that promotes and optimizes mass transport would be quite advantageous. We present a soft template approach for the routine fabrication of hierarchical multimodal nanoporous gold monolith (hm-NPG). This self-supporting framework composed of multimodal porosity is produced employing a synergistic mix of metal reduction, templating, annealing, and chemical dealloying. This method provides for the simultaneous optimization of active surface area and mass transport in a porous metal electrode. It is reliable, simple, economical, accessible, and environmentally friendly. The procedure should be scalable and can produce hm-NPG for use in applications such as biosensing, energy systems, biofiltration, and catalysis. The material visually displays two visibly unique structural length scales that range from the macroporous network structure (average pore size of 0.58 ± 0.29 μm) to the mesoporous pore/ligament morphology (average pore size of 37 ± 10 nm) as determined by SEM analysis. Modification by self-assembly with lipoic acid (LA) gave a coverage of 5.12 × 10^14^ molecules/cm^2^ of the hm-NPG surface, according to calculations made using thermogravimetric analysis (TGA) data. Following the dealloying procedure, a compositional study of the np-Au monolith using EDS revealed that it was almost 98.2 atomic % gold. The specific surface area of the hm-NPG was found to be 7.84 ± 0.01 m^2^/g (n = 3) through analysis utilizing the Brunauer–Emmett–Teller (BET) multi-point surface area method applied to krypton adsorption isotherms. BET analysis using N_2_ adsorption isotherms and the Barrett–Joyner–Halenda (BJH) pore distribution analysis gives strong evidence for the additional presence of micropores of diameter 2–3 nm, thus making the material likely trimodal.

## 1. Introduction

Recent developments of nanoporous gold materials with high surface area and a bicontinuous arrangement of mesoscale ligaments and interconnected void space have facilitated significant advancements in the fields of medicine, actuators, catalysis, and biosensing. These substances are produced using a free-corrosion method known as dealloying, which involves the selective dissolution of silver from silver/gold alloys [1,2]. The versatility, ease of use, and scalability of this sophisticated method of materials synthesis have experienced a boom in popularity. Additionally, the distinct morphological characteristics of nanoporous gold electrodes are closely related to their enhanced performance in a variety of electrochemical applications [3]. Despite these advancements, more work needs to be done in this area to create improved materials with high surface areas, as small pores restrict mass transport. To solve this problem, materials scientists are developing hierarchically porous gold [4]. It has become clear that hierarchical porosity control can greatly improve the performance of nanostructured metals for applications in electrocatalysis and chemical catalysis. The development of nanostructured metals has recently been largely driven by hierarchical design of porous systems. In addition to offering improved molecular accessibility as a novel class of hierarchical materials, hierarchically porous metals also offer distinctive continuous metallic frameworks for improving electron mobility [5,6].

Numerous cutting-edge synthesis techniques have been created in recent years to logically plan and control the pore sizes and architectures of hierarchically porous metals. There are two methods for incorporating and managing porosity in metals: templating and dealloying [7,8,9,10,11]. Templating creates pores with sizes between a nanometer and a micron using sacrificed inorganic or organic materials. To create a nanoporous material made of the more noble element, the dealloying process uses corrosion to selectively remove the least noble element(s) from an alloy [12]. Dextran was selected in our investigation as a soft, sacrificial template and reducing agent for the synthesis of the material presented here, referred to as hm-NPG (hierarchical multimodal nanoporous gold). Dextran’s water solubility made it easy to dissolve in concentrated aqueous solutions of metal salts, resulting in high metal loading. The aldehyde groups in dextran were used to reduce metal ions in situ and form metallic clusters. Finally, dextran was utilized to create macroporous frameworks and composites [13]. The dextran serves at least three important roles in the preparation; it helps to disperse the precursor salts, then it serves as a reducing agent and as a transitory template for mesopores and micropores. Our synthesis is suitable for the preparation of hierarchically structured meso/macroporous materials with adjustable macropore entrance size. It is possible that this method could be suitable for use in molds or patterns with further development. For the size-selective enrichment and separation of biomolecules with different sizes from complex systems, tuning the macropore size is crucial to the control of mass transport in the porous system [14].

Hierarchical bimodal nanoporous gold has been fabricated earlier by other researchers. Riedel et al. fabricated hierarchical nanoporous gold utilizing a complex nested network dealloying method; their fabrication method involves arc melting of Au-Ag alloy, followed by homogenization at 850 °C, and then cold rolling, a first dealloying step using 0.01 M sulfuric acid in a three-electrode setup, thermal coarsening and lastly a second dealloying step in 1 M perchloric acid [15]. Qian et al., in their work, fabricated hierarchical nanoporous gold by an electrochemical dissolution–disproportionation–deposition pathway in which metallic gold was dissolved from a smooth gold wire electrode in the form of AuCl_2_^−^, followed by disproportionation of Au(I) complexes and the redeposition of metallic gold onto the electrode surface, resulting in a 3D porous architecture [16]. Nyce et al. also reported another fabrication of hierarchical nanoporous gold; their method combines a hard templating step (using polystyrene spheres to form hollow Ag/Au shells) and a dealloying step in nitric acid [12].

Work on developing and studying hierarchical nanoporous gold has continued in recent years. The method of ultra-small-angle X-ray scattering was introduced and gave length scales for the upper and lower hierarchy levels of bimodal nanoporous gold that were consistent with determinations from SEM images [15]. The material was prepared from a master alloy of atomic composition Ag_90_Au_10_ that was subjected to a first electrochemical dealloying step to obtain a composition of Ag_75_Au_25_, followed by annealing at 300 °C, 400 °C, or 500 °C for 20 min. The final dealloying step was done electrochemically in 1 M HClO_4_. The mechanical properties of hierarchical nanoporous gold prepared by this method were studied and found to have improved compressive strength [17]. An electrodeposition strategy was used to produce hierarchical bimodal nanoporous gold on Au microelectrodes [18]. It was found that the presence of the larger pores resulted in enhanced diffusion from the bulk solution for electrochemical oxidation of ascorbic acid. The application of a two-step dealloying process to a ternary Au-Ag-Ge alloy in the form of round alloy crystals 2 mm in size was reported [19]. The ternary alloy of eutectic composition (Au-Ag)-Ge was first subjected to removal of Ge in a basic piranha solution to produce the upper hierarchy, followed by removal of Ag in 70% HNO_3_ to form the smaller pores. Using the hierarchical structure, the catalytic decomposition of H_2_O_2_ was enhanced by up to an order of magnitude compared to material formed by dealloying an Au-Ge alloy. The electrochemical dealloying and thermal annealing approach to produce hierarchical nanoporous gold was recently applied to give a series of samples of controlled pore size and study the transition from hierarchical to a unimodal structure using focused ion beam tomography [20].

The interest in hierarchical nanoporous gold arises from its potential advantages in applications in many fields, mainly due to the combination of high surface area and improved mass transport that it can provide over regular nanoporous gold of unimodal pore size distribution. In the field of catalysis, hierarchical nanoporous gold can provide improved catalyst utilization, higher limiting currents in electrocatalysis, and higher turnover rates for catalytic activity. Reactants will have improved access to inner pores, whether it be the case of solution-phase or gas-phase catalysis. Pore blockage close to the surface of the material will be less likely. If enzymes are being immobilized, then enzyme loading will likely be facilitated [21]. Although less work has been done, application of hierarchical nanoporous gold to biofiltration may also pose advantages of improved biomolecule capture due to the improved mass transport [22]. Biosensor electrodes based on hierarchical nanoporous gold should have improved analyte access to active sites of immobilized enzymes and other biomolecules. In energy-related applications, hierarchical nanoporous gold will be more easily modified with catalytic nanoparticles for use in hydrogen production, reduction of CO_2_, as fuel cell electrodes, as battery components, and as supercapacitors [23]. There are many potential research directions for this promising nanomaterial. Applications for unimodal nanoporous gold have been reviewed in detail [24,25,26].

In this study, we demonstrate the use of dextran as a soft template to synthesize a hierarchically structured nanoporous gold monolith with controllable pore sizes. Due to gold’s bioinert properties and ease of surface modification, it might be used in a variety of bioseparation processes with a high degree of selectivity. By carefully regulating the synthesis conditions, such as the alloy composition, the acid concentration, the amount of dextran, and the annealing temperature, hierarchically structured nanoporous gold monoliths with controllable pore sizes, high surface area, and large pore volume were successfully developed. To the best of our knowledge, dextran has not been reported as a sacrificial template and reducing agent for the fabrication of hierarchical nanoporous gold. Unlike some earlier methods [12,15,16], which require alloy arc melting, prolonged homogenization, multiple dealloying and annealing steps, electrochemical dissolution–redeposition processes, or hard-template fabrication using polystyrene spheres, the present approach employs a simple dextran-assisted soft-templating strategy followed by single annealing and a single dealloying step. We present evidence for the presence of micropores, mesopores and macropores, and the material thus appears to be trimodal; we refer to it as hierarchical multimodal nanoporous gold (hm-NPG). The hm-NPG fabrication process reported herein is simple, potentially more scalable, and utilizes an inexpensive, biodegradable, and biocompatible template. Furthermore, the larger pores combined with dealloying-generated nanopores provide a hierarchical architecture with enhanced mass-transport pathways while maintaining a high surface area.

## 2. Materials and Methods

### 2.1. Reagents

Nitric acid (trace metal grade) was from Fisher Scientific (Pittsburgh, PA, USA). Gold (III) chloride trihydrate, silver nitrate, dextran from *Leuconostoc mesenteroides* (average M_r_ = 60,000–76,000 Da), ethanol (HPLC/spectrophotometric grade), and α-Lipoic acid were obtained from Sigma–Aldrich (St. Louis, MO, USA). Milli-Q water (18.2 MΩ cm at 25 °C) was prepared using a Simplicity UV system from Millipore Corporation (Boston, MA, USA). All the chemicals were used as received. 14 carat yellow gold (1″ × 2″, 30 Ga, 0.25 mm thickness) was obtained from Stuller (Lafayette, LA, USA).

### 2.2. Instrumentation

ThermoFisher Scientific Apreo 2C field-emission scanning electron microscopy (Waltham, MA, USA) with ColorSEM and Energy Dispersive X-ray spectroscopy (EDS) was used to collect data on the surface morphology of the hm-NPG and NPG monolith material. Thermogravimetric analysis to estimate the SAM surface coverage was done using a Q500 thermogravimetric analyzer from TA Instruments (New Castle, DE, USA). A MicroActive 7.00 TriStar II Plus Version 3.04 Adsorption Surface Area and Pore Size Analyzer from Malvern Panalytical (Westborough, MA, USA) was used to analyze surface area and pore size of hm-NPG before and after dealloying, and regular NPG. Kr isotherms were collected for hm-NPG before and after dealloying, and for NPG. N_2_ isotherms were collected for hm-NPG. XRD was collected using a Bruker D8 Venture PHOTON II diffractometer (Madison, WI, USA) using Cu-Kα radiation of wavelength λ = 1.54178 Å. XPS data was collected using a Physical Electronics 5000 VersaProbe II spectrometer (Chanhassen, MN, USA) with a monochromatic Al Kα source. A VersaSTAT 4 potentiostat from Ametek SI (Oak Ridge, TN, USA) was used for electrochemical measurements.

### 2.3. Preparation of hm-NPG Monolith

Using a soft template technique and dealloying, a monolith with hierarchical architecture and apparently three unique ranges of pore size was produced. In Milli-Q water, stock solutions of 50 mM AuCl_3_ and 50 mM AgNO_3_ were prepared. A brown, viscous liquid was produced by combining dextran (2 g) in a solution of Au_10_:Ag_90_ (0.5 mL 50 mM AuCl_3_ + 4.5 mL 50 mM AgNO_3_), and thus 10:90 represents a mole ratio. This mixture was then left at room temperature overnight to allow the metal ions to be reduced. The paste gradually turned darker during this time because of the polysaccharide’s aldehyde groups partially reducing a portion of Ag(I) ions and the exposure of the paste containing AgNO_3_ solution to light, also causing the reduction of Ag(I) ions. After that, the composite was annealed for 30 min at 600 °C under air, which caused the organic template to be removed owing to its thermal degradation. The result was a porous alloy structure with larger-sized pores. After cooling to room temperature, a chemical dealloying step was carried out by immersing the annealed sample in concentrated nitric acid overnight to produce the smaller pores, followed by rinsing and centrifuging with Milli-Q water and ethanol [13,27]. The weight of the hm-NPG obtained after dealloying and vacuum drying was 6.5 mg. The initial mass of Au atoms in the starting solution is calculated as 7.6 mg, indicating a small fraction of Au lost somewhere in the process. This procedure was performed thrice to affirm the reproducibility of this hm-NPG fabrication. The hm-NPG monolith was fragile and could be easily crushed into grains. A picture of the as-fabricated hm-NPG in an Eppendorf pipette is shown in Figure S6. The product material is about 4 mm in diameter and has the shape of a small pellet.

### 2.4. Preparation of Nanoporous Gold from Yellow Gold Plate

Nanoporous gold with unimodal pore size distribution was prepared for comparison purposes following the procedure reported by Tan et al. [28]. In short, a 14 carat yellow gold plate with thickness 30 Ga (0.25 mm) and dimensions 1.0 inch × 2.0 inch was purchased from Stuller (Lafayette, LA, USA), with weight percent composition of the alloy stated as 58.5% Au, 5.0% Ag, 31.60% Cu, and 4.9% Zn. Fabrication of the NPG monolith began by cutting the gold alloy into pieces (2.0 mm × 2.0 mm). This was then followed by dipping in concentrated nitric acid for 48 h, with the renewal of the nitric acid every 12 h. This was done to etch out the less noble metals present in the yellow gold alloys, leaving behind just the gold with numerous pores formed because of dealloying. The resulting plates were thoroughly cleaned with Milli-Q water and ethanol to completely remove the acid; the plates were then placed under vacuum overnight and then crushed into grains. The nanoporous structure of the NPG was confirmed by SEM, and its surface elemental composition before and after dealloying was confirmed by EDS (Figures S1 and S2). Both NPG plates and our hm-NPG material, as prepared in one piece, can be crushed into micron-sized particle fragments.

The composition of the yellow gold reported by the manufacturer is in weight percent. In contrast, EDS measures atomic composition in percentage of each type of atom. A weight percent composition of 58.5% Au, 5.0% Ag, 31.6% Cu, and 4.9% Zn corresponds to an atomic percent composition of 32.4% Au, 5.1% Ag, 54.3% Cu, and 8.2% Zn. There is little reason to suspect that the composition given by a reputable supplier of basic materials for making jewelry is incorrect. EDS is a surface-sensitive method, and the composition of an alloy at the surface can be different from the bulk due to preferential segregation of some metals to the surface due to their lower surface energy [29]. EDS does not give a true weight percent for the entire sample; it only probes the surface to a certain depth that is dependent on the accelerating voltage and element being detected. The weight percents reported from EDS apply only to the sample regions inside the interaction volume with the electron beam. For the 15 kV voltage used in EDS in this study, a penetration depth of 1–3 μm is expected, and the thickness of the 14-carat gold alloy used here is 250 μm, so less than 1% of the sample is being probed. It is reasonable to expect enrichment of Au to the surface, and 40% atomic percent (40 at. %) is quite plausible due to the lower surface energy and higher oxidation potential of Au compared to Cu. The same considerations apply to the behavior of Zn in the alloy [30]. Some studies have reported the comparison of the bulk composition with the surface composition of metal alloys, although a study of 14-carat gold alloy does not seem available.

### 2.5. Structural and Chemical Characterization of hm-NPG and NPG Monolith: SEM, Energy-Dispersive X-Ray Analysis, X-Ray Diffraction (XRD) and X-Ray Photoelectron Spectroscopy (XPS)

SEM was used to analyze the surface morphology of the fabricated hm-NPG before and after dealloying and the NPG monolith. To describe the morphological aspects, grains of the crushed monolith were put on the SEM stage. Energy-dispersive X-ray spectroscopy (EDS) was used for the elemental analysis, with the voltage and current set to 15 kV and 1.6 nA, respectively. XRD was used to determine the purity and crystallinity of the fabricated nanoporous material; the data was collected using a Bruker D8 Venture PHOTON II diffractometer using Cu-Kα radiation of wavelength λ = 1.54178 Å. The intensity data was collected and integrated using the Bruker APEX6, (Madison, WI, USA) (with XRD2–Eval Plug-In) program suite. The intensity data was collected over a 2θ range of 10° to 90° with a step size of 0.05°. The elemental analysis and the oxidation states of the fabricated hm-NPG monolith was further confirmed by XPS using a Physical Electronics 5000 VersaProbe II spectrometer with a monochromatic Al Kα source.

### 2.6. Evaluation of Specific Surface Area of hm-NPG and NPG Monolith: Krypton BET Analysis

Specific surface area analysis was performed on a MicroActive 7.00 TriStar II Plus Version 3.04, using krypton adsorption at 77 K, as the surface areas of the materials are ≤5–10 m^2^/g; therefore, krypton with significantly lower vapor pressure (P_0_) than N_2_ minimizes dead space or dead volume errors, resulting in better sensitivity, making the result more accurate. Krypton isotherm analysis is often employed for porous materials with surface areas low enough to make acquiring good N_2_ isotherm data challenging or not possible [31]. Krypton isotherm data does not allow for the BJH (Barrett–Joyner–Halenda) pore size analysis [32]. The application of the BJH analysis requires a full adsorption–desorption isotherm for application of the Kelvin equation, and the amount of krypton that condenses is insufficient, with krypton pore condensation reported to vanish for pores above 6 nm [33]. The Kr BET analysis was performed on the Au-Ag alloy after annealing of the alloy paste, on the hm-NPG monolith resulting from dealloying of the Au-Ag alloy, and on the NPG fabricated by dealloying yellow gold in nitric acid. Annealing was done using a Barnstead Thermolyne 47900 digital lab furnace (model F47915) (Dubuque, IA, USA. The same Tristar instrument was used to acquire N_2_ BET data on a sample of hm-NPG to acquire a full adsorption–desorption isotherm cycle and perform a BJH analysis and gain information on the pore size distribution.

### 2.7. Surface Coverage of Lipoic Acid Molecules on Monolith: Thermogravimetric Analysis

A self-assembled monolayer (SAM) of lipoic acid was created by immersing 4 mg of hm-NPG monolith material in 1000 μL of 1 mM lipoic acid in ethanol. After being left for around 17 h (overnight), the monolith particles were washed three times with ethanol and dried in vacuum. The dried hm-NPG was then placed in a platinum weighing pan and was heated inside a thermogravimetric analyzer from ambient temperature to 550 °C at a ramping rate of 20 °C min^−1^. Nitrogen served as carrier gas and was passed at a 40 mL min^−1^ flow rate. N_2_ gas was allowed to pass through the sample for 5–10 min before the temperature ramp began. The analysis produced the initial mass, mass losses, and % weight change. Based on the net mass loss and the surface area identified by BET, the surface coverage of lipoic acid on hm-NPG surfaces was estimated.

### 2.8. Data Analysis

Using OriginLab, all the data calculations and graphing were completed. ImageJ version 1.54s (imagej.nih.gov/ij/) was used to analyze the ligament width, pore diameters, and inter-ligament distances.

## 3. Results and Discussion

In porous materials, improving mass transfer and increasing the active surface area are mutually exclusive processes. By creating continuous channels at various length scales, structural hierarchy could resolve this conflict. Making porous metallic materials with distinct hierarchical levels, however, is quite challenging. A hierarchical multimodal nanoporous gold (hm-NPG) monolith with trimodal hierarchy is prepared here using a simple technique combining chemical dealloying and soft templating. The current method has potential for producing further hierarchically porous metals with improved structural and functional qualities [34].

### 3.1. The Hierarchical Multimodal Pore Structure Characterization of hm-NPG and NPG Monolith

**Figure 1:**
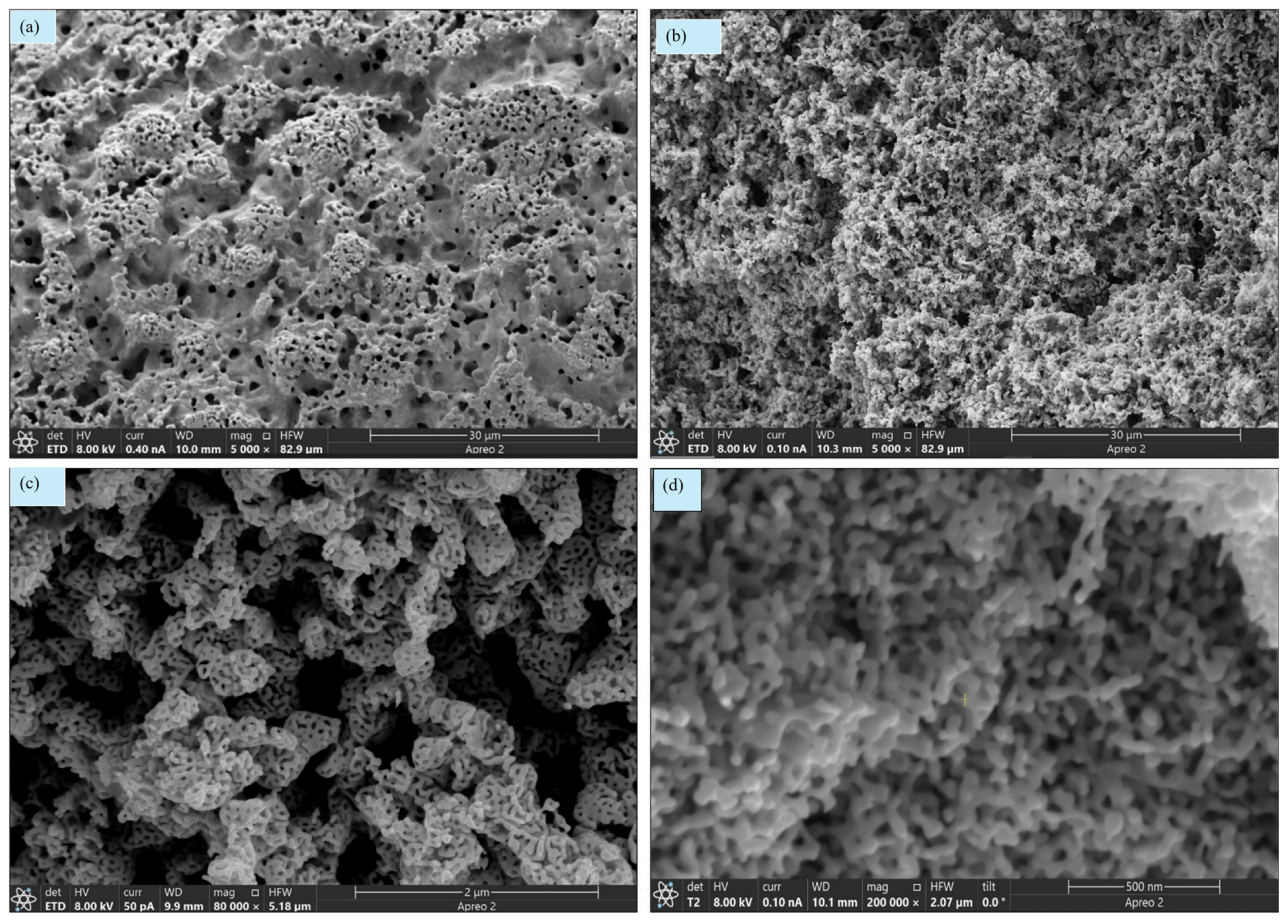

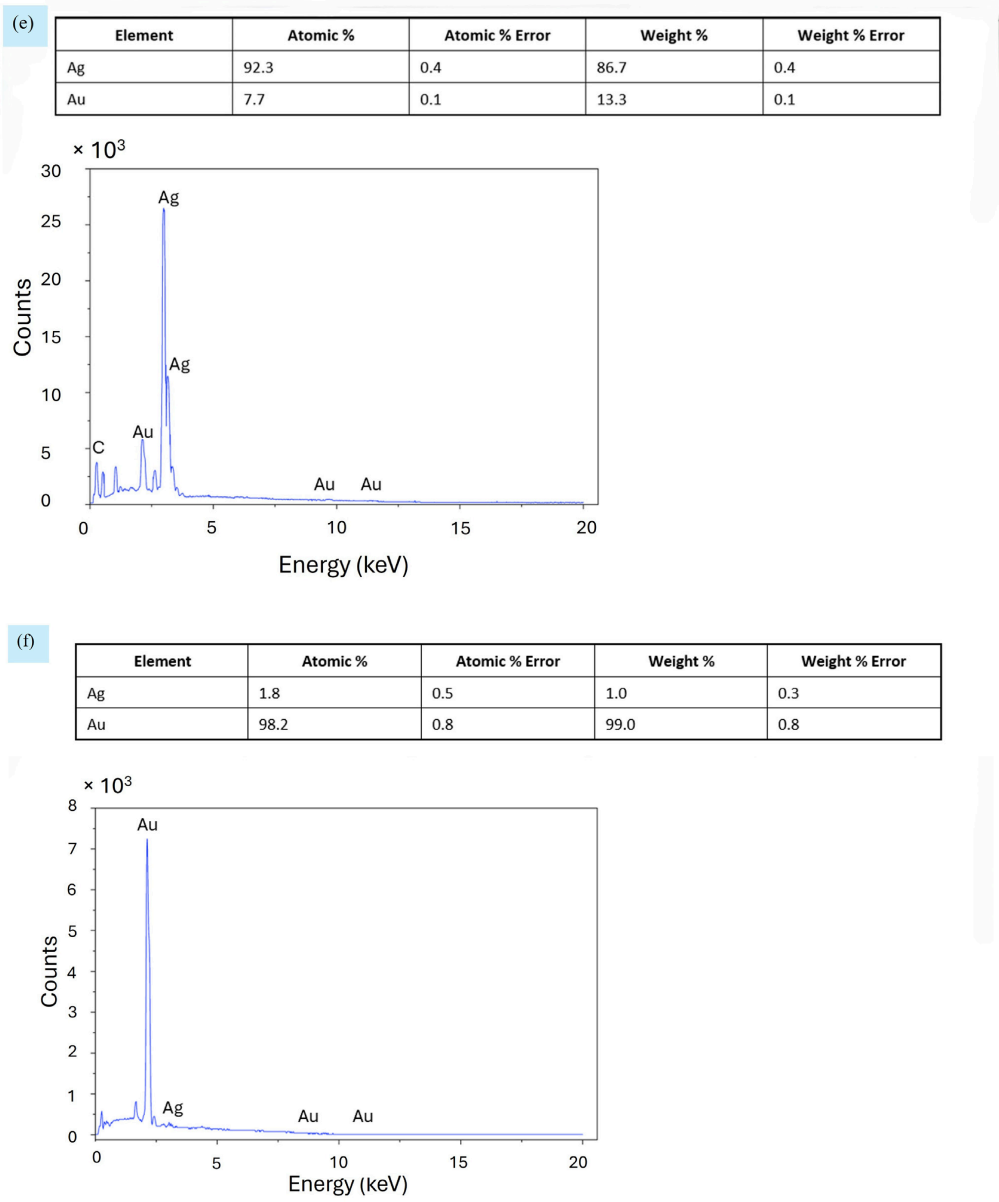
SEM images of (**a**) gold–silver alloy monolith after being annealed at 600 °C showing pore coarsening, (**b**) pores of upper hierarchy, (**c**) pores of lower hierarchy after chemical dealloying, (**d**) Nanoporous gold monolith, (**e**) EDS data after annealing at 600 °C, and (**f**) EDS data after chemical dealloying in concentrated trace metal grade nitric acid.

To understand the morphology, porous structure, and the function of residual silver, SEM and compositional analyses were carried out at each stage of the preparation. It has been observed that the rise in compaction of the sponge-like framework caused by the coarsening of the linked silver particles, development of the sintering necks, and shrinkage of the empty spaces was related to the change in porosity [13]. After heating the mixture of gold, silver, and dextran to 600 °C, the organic template was destroyed thermally and the metal ions reduced, leaving behind an entire silver-gold alloy monolith with only the larger pore sizes visible (Figure 1a). The existence of the hierarchical porous structure, which consists of multiple micro/mesopores and interconnected macropores, has been confirmed by SEM micrographs (Figure 1b,c). The 3D gold nanostructured monolith was found to have a uniform distribution of the multimodal porous architecture wherein silver was dissolved/etched with concentrated HNO_3_, forming voids in the framework. Using EDS, we examined the percentage composition of Au and Ag atoms after annealing (Au-Ag alloy), EDS showed that there was about 92.7% Ag and 7.3% Au present in the alloy (Figure 1e), upon chemical dealloying of the Au-Ag alloy nanomaterial with concentrated nitric acid, EDS examination revealed that there was only 1–2 atomic% of silver remaining, which is much less than the starting composition of the alloy (Figure 1f). We further corroborated this with a ColorSEM image in Figure S5. The SEM image of traditional NPG without hierarchy used for comparison is shown in Figure 1d.

**Figure 2:**
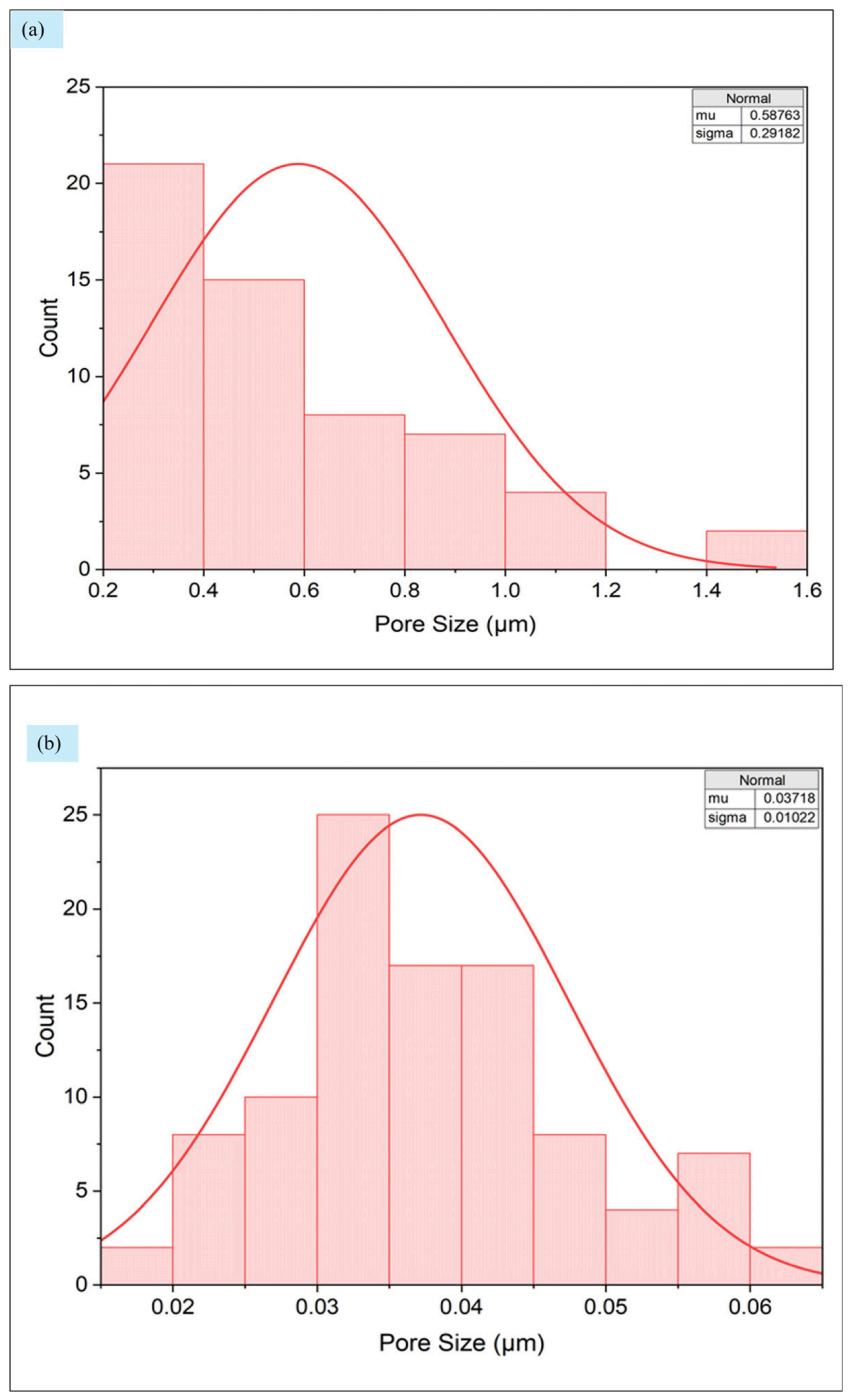

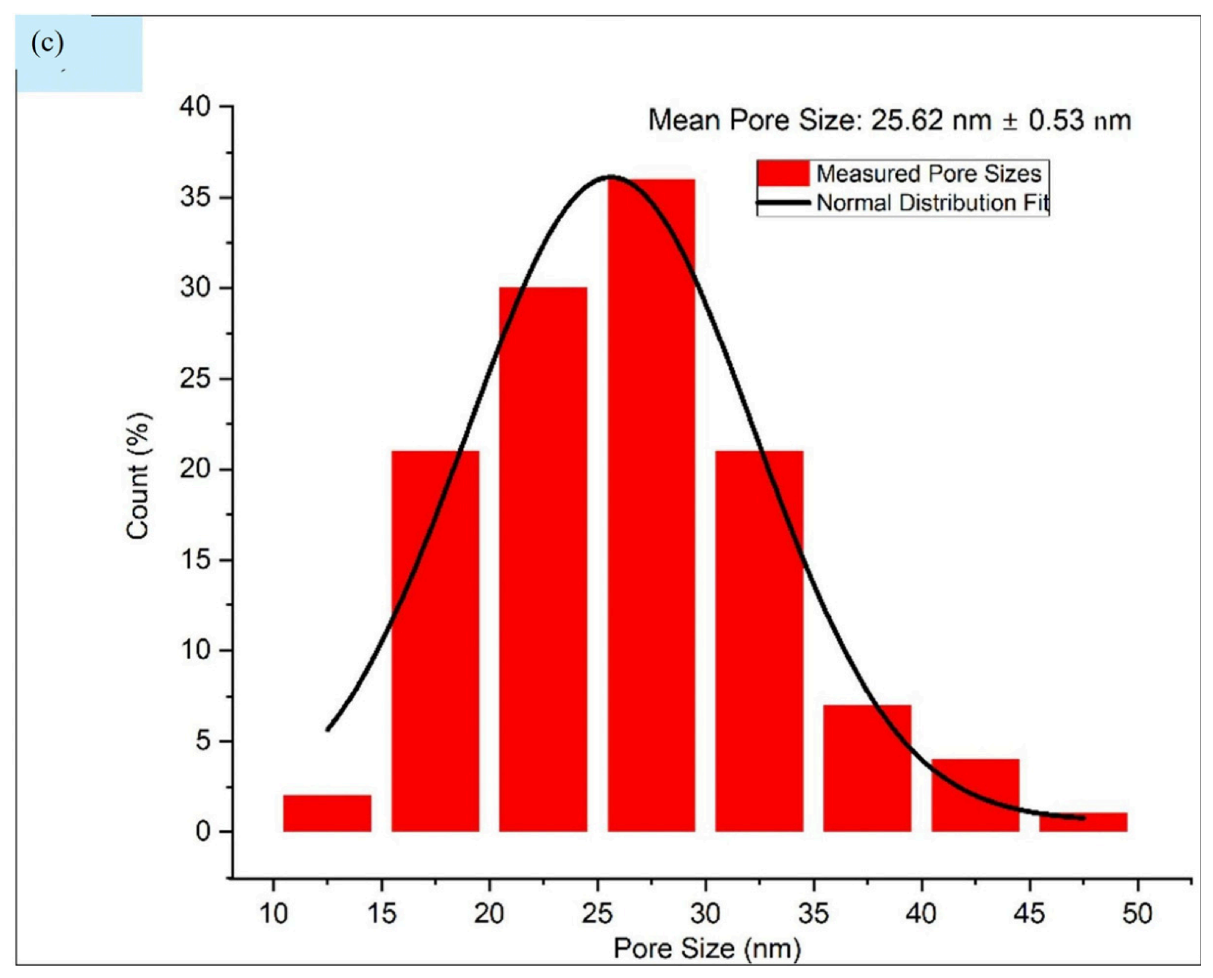
Pore size distribution in hierarchical multimodal nanoporous gold monolith shown as histograms based on the measuring interligament gaps for the indicated number of counts from SEM images, representing (**a**) upper hierarchical pore size distribution and (**b**) lower hierarchical pore size distribution, and (**c**) pore size distribution in unimodal NPG. The solid lines represents fits of the histogram to a normal distribution, as used to determine a standard deviation.

The resulting hierarchical nanoporous gold material contained pores of upper hierarchy that measured 0.58 ± 0.29 μm and an open framework comprising metallic gold ligaments that were connected and had discrete crystallites (Figure 2a). The distribution of pore size for the lower hierarchy was found to be 37 ± 10 nm (Figure 2b). To create the hierarchical nanoporous gold, the viscous dextran matrix had to be broken down and transformed into a carbon foam that contained metallic particles. Upon gradually removing the carbon by heating to higher temperatures, the material was compressed, and the metallic particles fused to make an open framework sponge-like material. This material was then treated to chemical dealloying to produce the hierarchical pore structure [13]. The fabricated NPG has a measured pore size of about 25.6 ± 0.53 nm (Figure 2c). The EDS of flat yellow gold before and after dealloying is shown in Figure S1, so as to confirm the almost complete dealloying of the yellow gold and pore formation due to etching away of the Cu, Zn, and Ag metals present in the commercial alloy. Again, this is reported with the caveat that EDS is a surface-sensitive method and not a bulk characterization.

To confirm the reproducibility of the hm-NPG fabrication (pore size for the upper and lower hierarchy) we repeated the fabrication two more times, followed by the measurement of the pore sizes; for sample 2, the pore size was measured to be 0.63 ± 0.22 μm (upper hierarchy) and 41 ± 12 nm (lower hierarchy), and for sample 3, the pore size was measured to be 0.46 ± 0.14 μm (upper hierarchy) and 37 ± 12 nm (lower hierarchy). The close agreements in the measurements confirm that the hm-NPG fabrication technique described herein is reasonably reproducible and consistently yields comparable pore dimensions in independently prepared samples. The SEM images and the pore size measurements for the two other samples can be found in Figure S3 of the Supplementary File.

**Figure 3:**
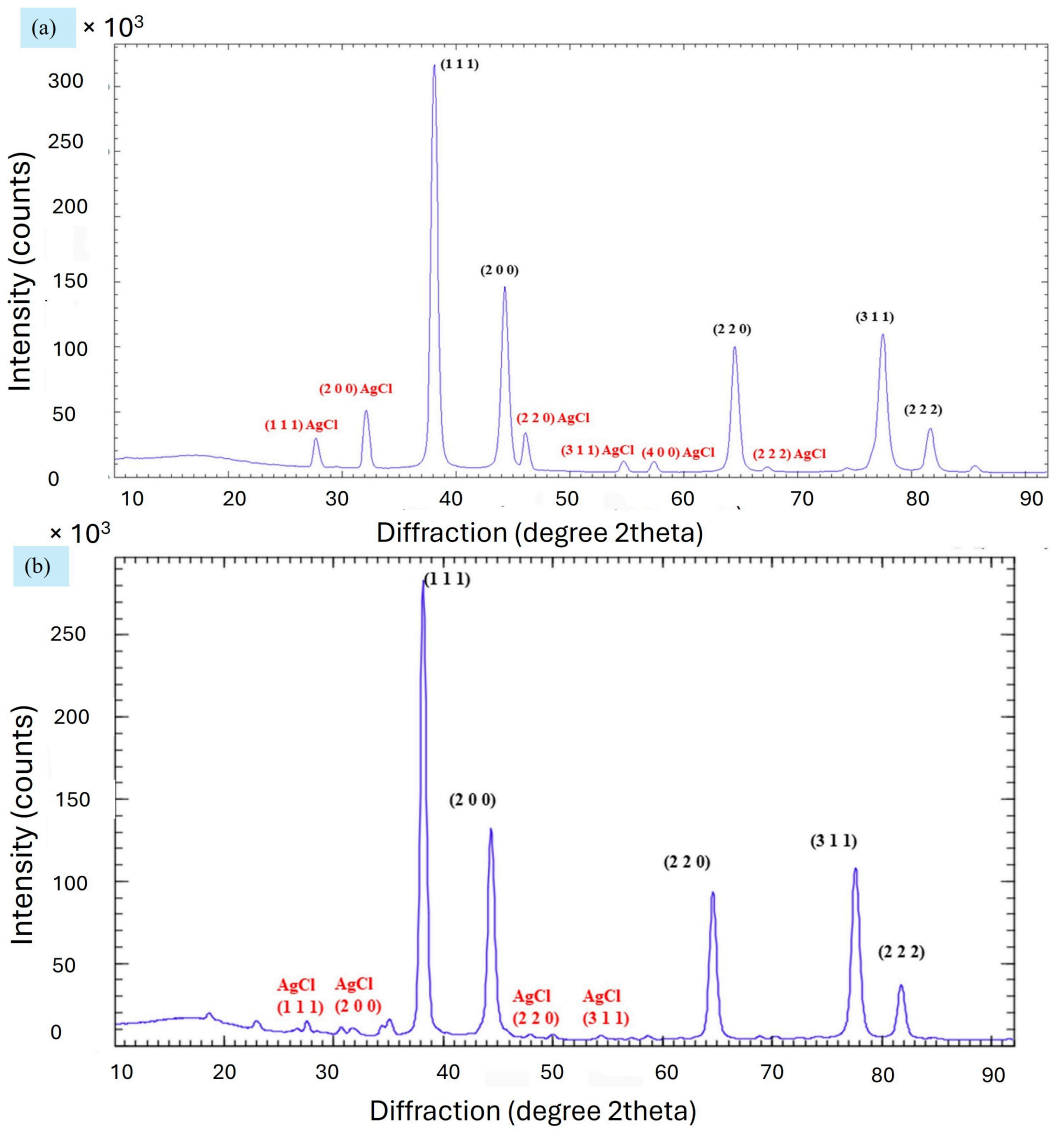
XRD analyses of (**a**) Au_10_Ag_90_ alloy after annealing at 600 °C and before dealloying in nitric acid and (**b**) after chemical dealloying in nitric acid.

The crystal structure of the Au-Ag alloy and hm-NPG was determined by powder X-ray diffraction (XRD) analysis. Figure 3a and Figure 3b show the XRD pattern of the Au_10_-Ag_90_ alloy after annealing at 600 °C and before dealloying in nitric acid and after chemical dealloying in nitric acid, respectively. Distinct strong diffraction peaks at scan angles 2θ of 38.08°, 44.26°, 64.39°, 77.33° and 81.47° are observed, and these index the (111), (200), (220), (311) and (222) hkl planes respectively for the Au-Ag alloy (Figure 3a). The peak positions and the planes correspond to the face-centered cubic (fcc) structure of Au-Ag alloy. Similarly, from Figure 3b, the intense diffraction peaks of hm-NPG at angles 2θ of 38.15°, 44.35°, 64.54°, 77.53° and 81.68°, indexing the (111), (200), (220), (311) and (222) hkl planes, respectively, for Au nanoparticles. It is understood that, in the case of a bimetallic Au-Ag alloy, the peak positions (2θ, dhkl) corresponding to the lattice planes are similar to those for the monometallic Au and Ag nanoparticles, since the Au and Ag metals have similar lattice constants, such as 4.077 and 4.078, respectively. It is clear from the above observation that there is a slight shift in the 2θ (peak positions) values of (200), (220), (311) and (222) planes with respect to hm-NPG when compared to the Au-Ag alloy [20,21], which is attributed to the conversion of the Au-Ag alloy to pure Au material. Also, the peak positions of the hm-NPG are in strong agreement with the Au-XRD pattern of the JCPDS database [PCPDFWIN-PDF652870]. The sharp, intense peaks show the crystalline nature of the Au-Ag alloy and hm-NPG; the prominent peak at 2θ = 38.15° reveals that the crystalline growth is dominated along the (111) plane and the preferred orientation is along the (111) direction, which are characteristics of an fcc crystal structure. Some of the smaller peaks in the final hm-NPG can be indexed to AgCl, which appears to be present in a very small amount. There are two very small peaks at the lowest angles that remain unidentified. In conclusion, the XRD pattern in Figure 3b is the characteristic pattern of pure Au nanocrystals with a slightly distinct crystalline structure; however, it retained the expected planes present in the alloy after chemical dealloying in nitric acid [35,36]. The indexed powder diffraction peaks of Au-Ag alloy and hm-NPG are given in Tables S1 and S2 of the Supplementary File.

The Scherrer equation is used to estimate the size of crystalline domains in a material by analysis of broadening of diffraction peaks. The Scherrer equation is D = K λ/β cos θ, where D is the crystalline domain size, K is a shape factor often assumed to be 0.9, λ is the X-ray wavelength, b is the full width at half-maximum (FWHM) of a diffraction peak given in radians and θ is the Bragg angle. The equation assumes that peak broadening is only associated with crystallite size. The equation is often applied to nanoparticles and can determine if nanoparticles are polycrystalline or single-crystal [37]. Peak broadening is typically significant only up to 100 nm. The average grain size found for the initial Au/Ag is 13.9 ± 0.8 nm, and 14.4 ± 1.1 nm for the final dealloyed material. The Scherrer equation has been applied in several studies of nanoporous gold [38,39]. However, since the structure of nanoporous gold is one of interconnected ligaments superimposed over any finite-size crystallite arrangements, interpretation of the Scherrer equation for nanoporous gold has been noted as an estimation [38]. In the reported study, a value of D = 37.2 ± 1.2 nm is obtained, and it was concluded that ligament size and microstrain were the two sources of peak broadening and the ligament structure obviates lattice coherency as a source of peak broadening. The observation of a smaller value of D here for hm-NPG could be speculated as arising from the presence of micropores reducing the size of coherent regions.

**Figure 4:**
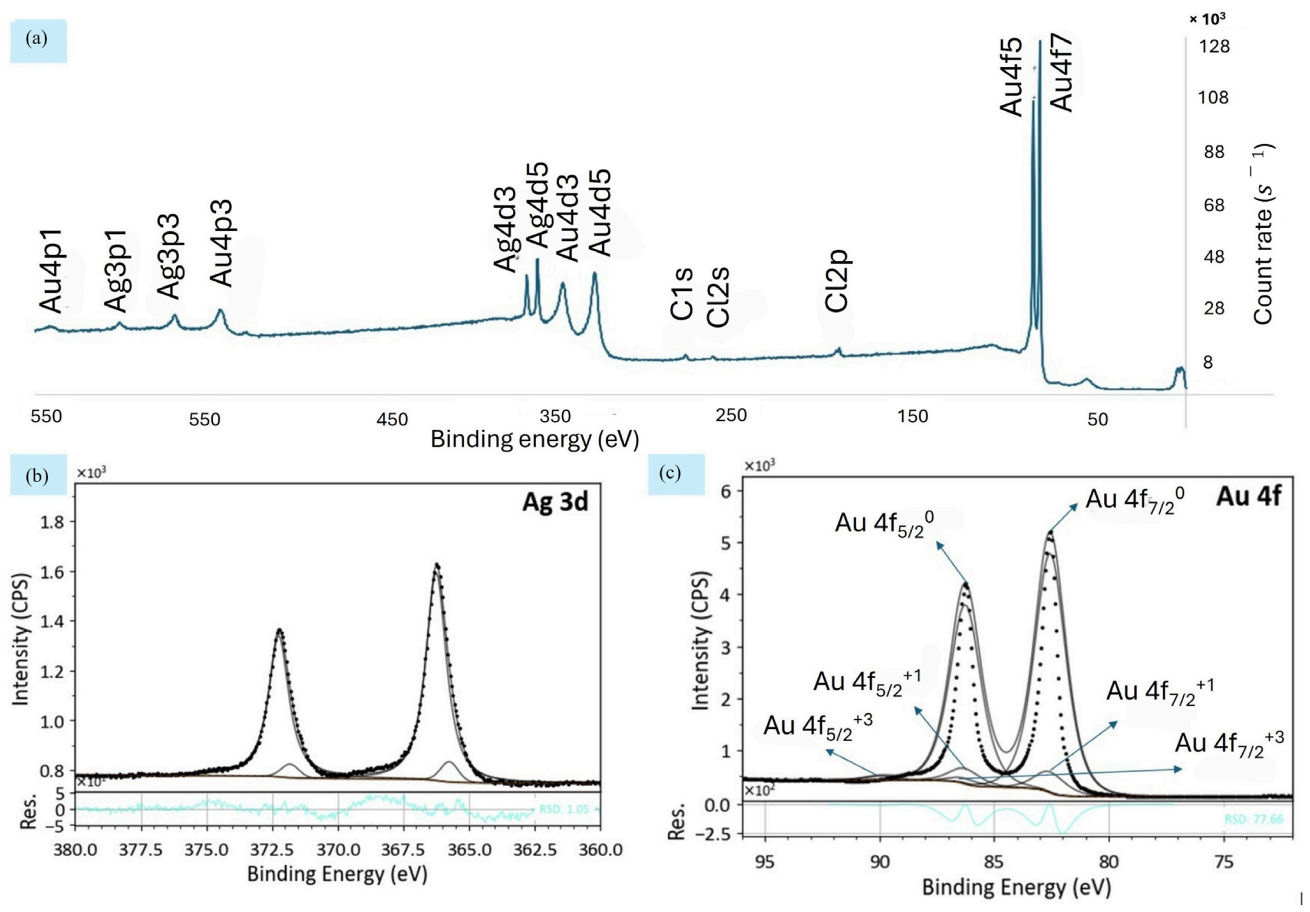
(**a**) XPS sample survey spectrum of hm-NPG, (**b**) high-resolution Ag 3d spectrum and (**c**) high-resolution Au 4f spectrum. In (**b**,**c**), the small lower panel represents residuals from the data fits for the high resolution spectra.

X-ray Photoelectron Spectroscopy (XPS) was used to study the elemental composition of hm-NPG in this work. The survey scan in Figure 4a shows the presence of binding energy peaks for Au 4f, Au 3d, Ag 3d, Cl 2p, and C 1s, indicating the presence of Au, Ag and Cl, and C in the hm-NPG. The high-resolution Ag 3d spectrum in Figure 4b displays two weak peaks at 365.74 eV and 371.81 eV, corresponding to the Ag 3d spin–orbit components with an intensity ratio of 3:2. In the same way, the high-resolution Au 4f spectrum in Figure 4c possesses a characteristic doublet at 82.55 eV and 86.25 eV corresponding to Au4f_7/2_ and Au4f_5/2_, separated by ~3.7 eV. This is indicative of zero-valent gold in the hm-NPG, confirming the presence of gold in its elemental form. The peak observed at ~198–200 eV corresponds to the Cl 2p region as labeled in the XPS sample survey in Figure 4a. This is characteristic of chloride ions, which may confirm the presence of a trace amount of AgCl as observed in the XRD data of Figure 3b. A small bump around ~530 eV may be attributed to O1s, which may be from some surface oxide formed when oxygen reacts with trace leftover Ag (Ag_2_O) or a trace amount of some other oxygen-containing species.

### 3.2. BET and BJH Analysis

**Figure 5:**
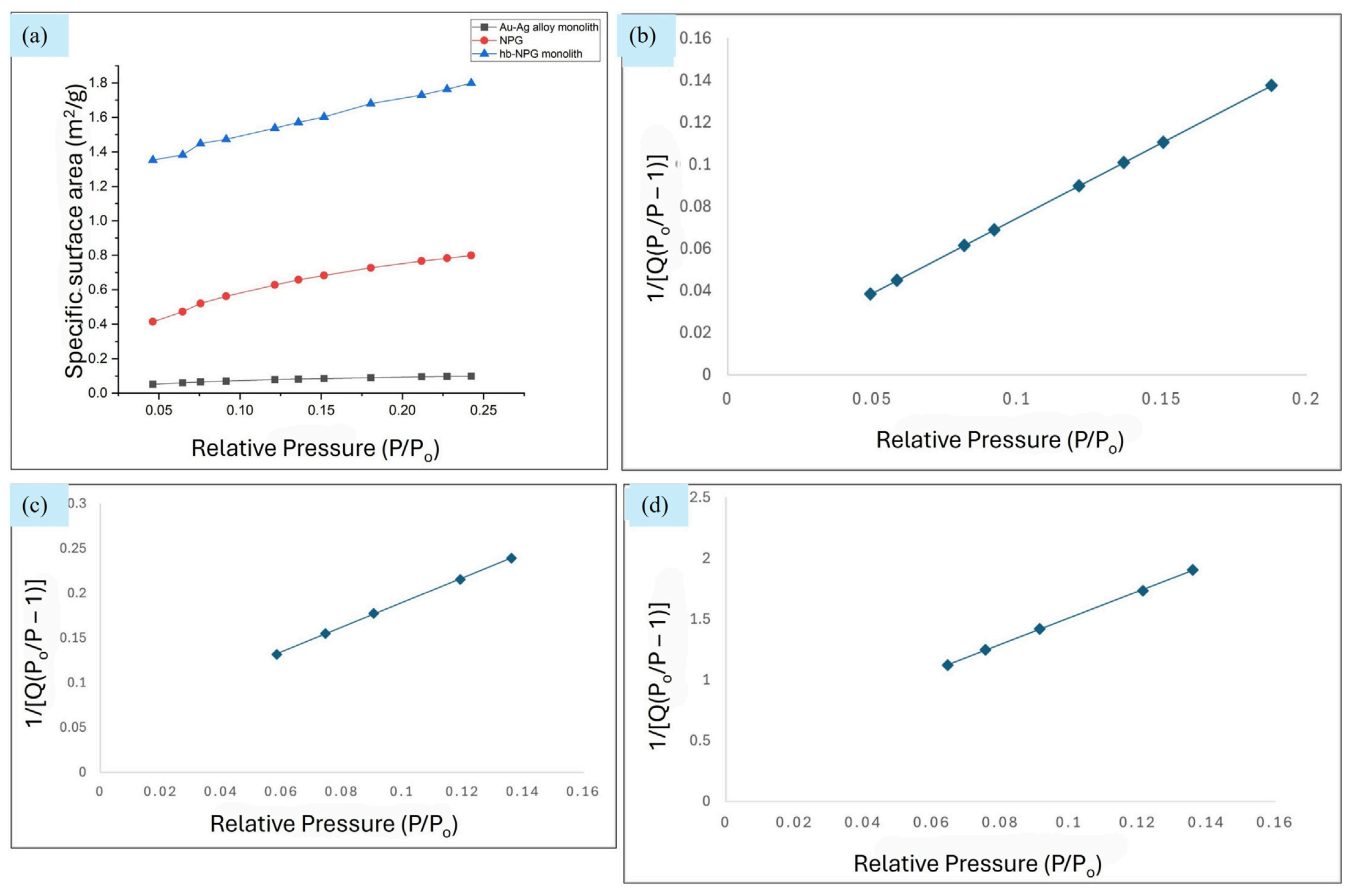

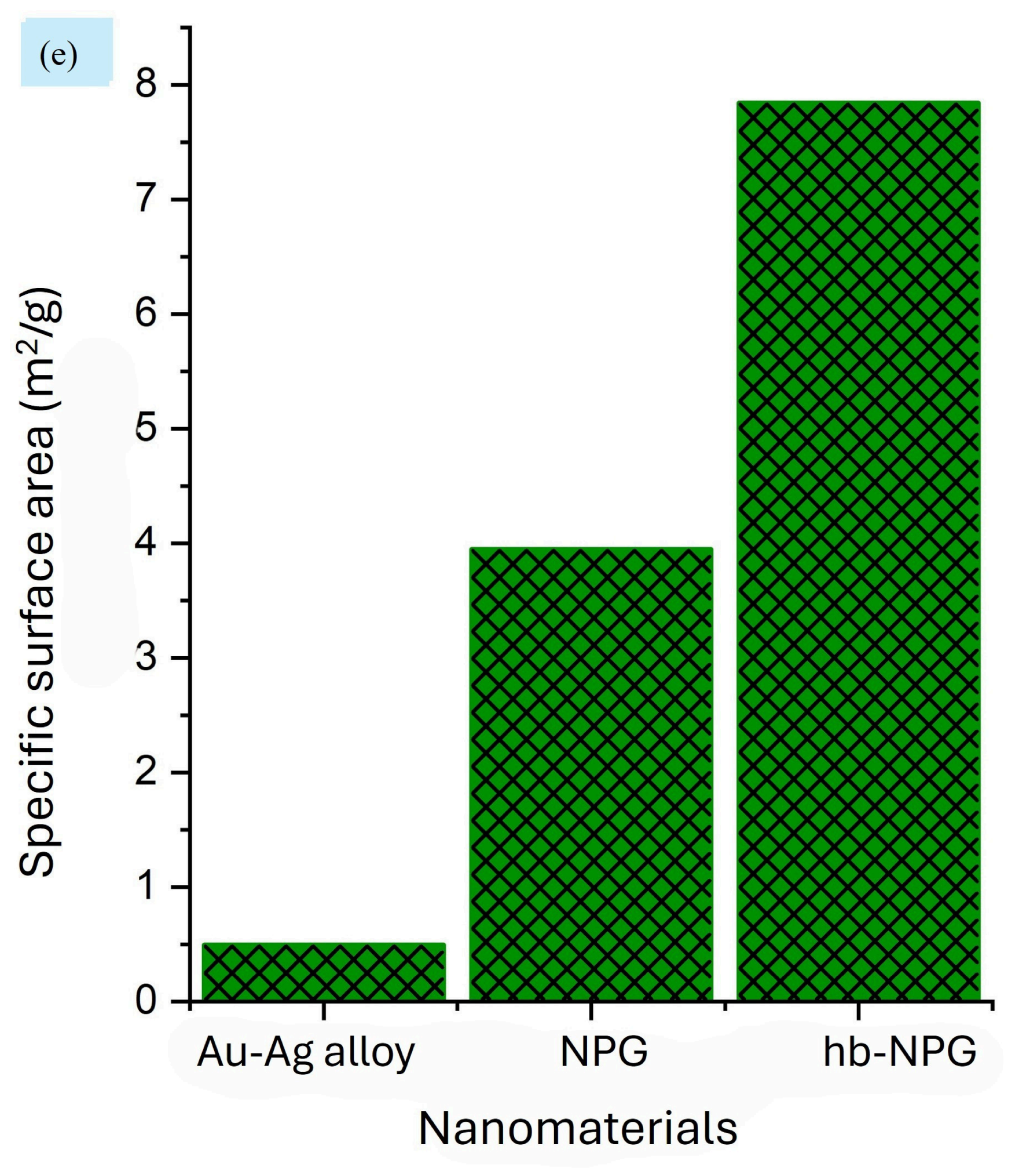
BET isotherm (Kr gas) (**a**) for hm-NPG monolith, NPG, and Au-Ag alloy. BET specific surface area plot of (**b**) hm-NPG, (**c**) NPG, (**d**) Au-Ag alloy, and (**e**) bar chart depicting the surface areas of each material.

BET analysis by krypton gas adsorption was used to determine the surface area of the Au-Ag alloy monolith formed after annealing, the hm-NPG monolith formed from the dealloying of the Au-Ag, and regular NPG. Krypton adsorption at 77 K is considered the most accurate gold standard for measuring the surface area of materials with lower surface areas. The specific surface areas for the Au-Ag alloy after annealing and before dealloying, NPG and hm-NPG, were calculated from the BET plots using the relevant adsorption isotherms [40], as shown in Figure 5a. The BET surface area plot is also known as the multipoint surface area, calculated with three or more pressure points, as seen in Figure 5b–d (hm-NPG, NPG and Au-Ag alloy respectively). We also depicted the surface area measurements for each of our materials in Figure 5e. There exists, between the materials, a great difference in specific surface area; hm-NPG shows a considerably higher surface area of 7.84 m^2^/g, as compared to NPG, which gave a surface area of 3.95 m^2^/g, and the initial Au-Ag alloy having a surface area of 0.50 m^2^/g, which is so small it could only be measured by Kr BET analysis. These results suggest that mesopores were present in the folds on the monolith’s skeleton frame surface, increasing the surface area and number of exposed sites. The fabricated monolith demonstrated a higher surface area and a hierarchically porous structure that not only provided pathways for fluid transport or molecular diffusion, but also additional sites and surface to promote interaction between an analyte in solution [41]. There also exists a clear relationship between the type of material (porosity) and the surface area obtained by BET.

**Figure 6:**
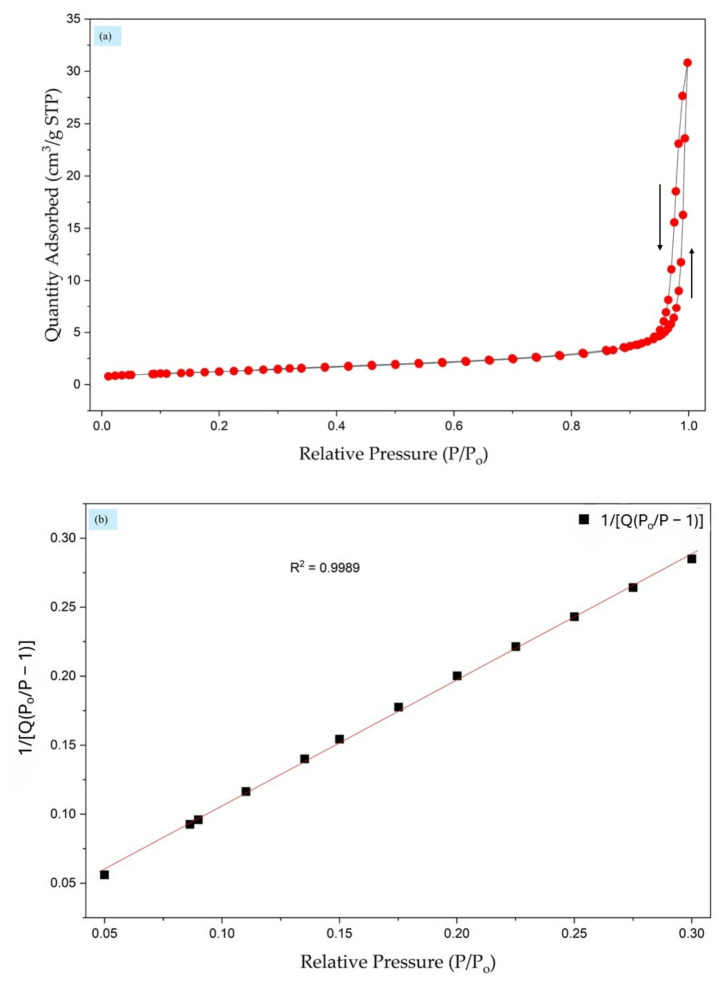

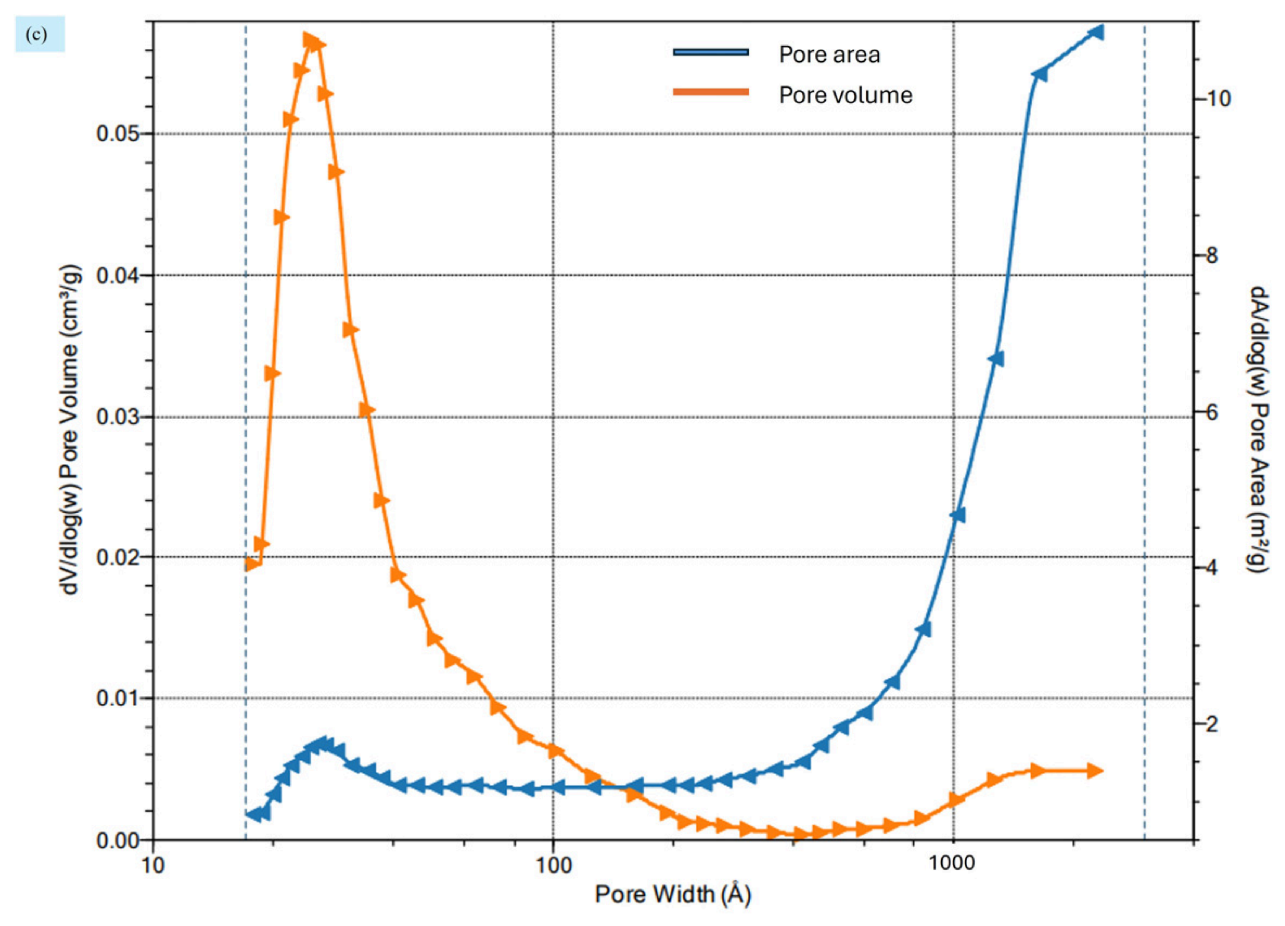
N_2_ physisorption characterization of hm-NPG: (**a**) N_2_ adsorption–desorption isotherm obtained at 77 K, showing both adsorption (up arrow) and desorption (down arrow) branches. The red dots are the data points and the black line simply connects then to show adsorption followed by desorption (**b**) BET fitting plot used to obtain the specific surface area, and (**c**) BJH pore size distribution graph showing dV/dlog(w) for pore volume and dA/dlog(w) for pore area as a function of pore width.

N_2_ BET data provides important insights into the pore structure of hm-NPG. Figure 6a shows the adsorption–desorption isotherm for N_2_ on hm-NPG. The isotherm is of type IV, confirming the presence of mesopores. The isotherm shows a small hysteresis loop of type H1, indicating a rigid material with mesopores and rapid capillary condensation and evaporation inside the pores. The BET surface area obtained is 4.69 ± 0.05 m^2^/g. Figure 6b shows the BET plot for the fitting range up to P/P_o_ = 0.300. The BET ‘C’ value is 62.9, and values between 50 and 150 indicate validity of the BET model and that the first monolayer of gas molecules adsorbed before the initiation of multilayer formation [42]. Figure 6c shows the BJH pore size distribution plot as dV/d(log(w)) with pore volume in cm^3^/g versus pore width in Å. There is a peak near 25 Å, and this indicates a significant population of micropores of size ~2–3 nm. The plot is steady over a middle range of pore widths and then increases dramatically towards pores greater than 100 nm. The plot of dA/d(log w), where A is pore area, shows that the pores in the 2–3 nm range make a very significant contribution to the surface area of the material. The average pore diameter is 40.7 nm for adsorption, and the average desorption pore diameter is 1.4 nm. This result is typical for nanoporous gold and is attributed to the ‘ink bottle’ pore effect when wider pores are trapped behind narrow channels. The average pore diameter of 40.7 nm indicates that a wide range of pore diameters is being averaged, from micropores, mesopores and some macropores. The micropores are too small to observe by SEM, but our SEM images do show the mesopores and some macropores. In combination with the SEM images that show mesopores and macropores, this data provides strong evidence for a population of micropores and that the material can likely be considered as trimodal, consisting of all three types of pores. The BJH analysis in our prior study of dealloyed 10-carat gold plates had a very different outcome, and the peak in the pore size distribution was around 90 nm and there was no evidence for the presence of micropores [28]. To our knowledge, this is the first report for a nanoporous gold material to include both Kr and N2 BET data. A significant difference is seen in the specific surface area outcome for the two methods (N_2_ BET gave a specific surface area of 4.69 m^2^/g). Kr BET is considered more accurate for specific surface area, but only N_2_ BET affords the possibility of conducting a BJH analysis.

### 3.3. TGA

The change or rate of change in a material’s weight as a function of temperature can be quantitatively measured using TGA and used to quantify the loading of organic molecules while considering the specific surface area of the material. TGA was used to evaluate the number of lipoic acid molecules loaded onto the hm-NPG monolith. We conducted pyrolytic decomposition on the air-dried modified sample in an inert environment while scanning up to 550 °C. Lipoic acid molecules were anticipated to totally break down before this temperature [27].

TGA was used to analyze the thermal stability and loading capacity of the monolithic material (before and after dealloying). A significant weight loss happened as the temperature increased from 150 to 350 °C in the lipoic acid-loaded dealloyed sample from which the hm-NPG was created. Due to the formation of bigger pores in the gold–silver alloy after the stage of annealing, the starting temperature of the weight loss during the TGA scan for the lipoic acid-loaded annealed sample gradually changed from 200 to 400 °C. It is evident that the lipoic acid loading in the dealloyed monolith was higher than in the annealed sample [41].

**Figure 7:**
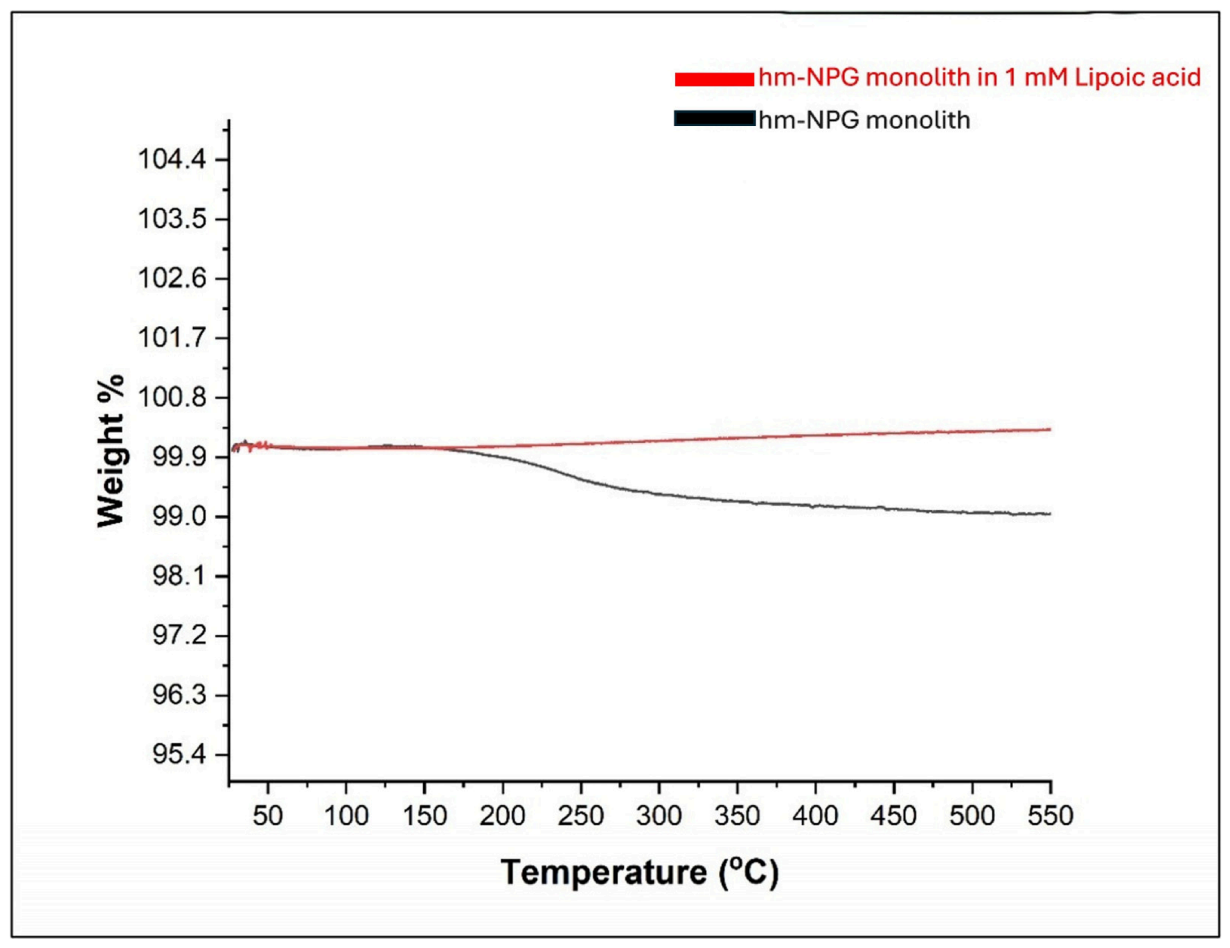
Thermogravimetric curve of hm-NPG without LA (red) and hm-NPG with LA (black).

It was found that the sample with lipoic acid (LA) immobilized on hm-NPG monolith had lost 0.82% of its original weight throughout the temperature scan. The average mass loss for the hm-NPG was determined to be 0.03 mg (the initial average sample weight was 3.29 mg). This mass loss amounted to $1.45\times 10^{-7}$ moles (molecular weight of LA is 206.33 g/mol) of self-assembled LA on the sample surface, equivalent to $8.73\times 10^{16}$ molecules $\left(10^{18}\;{molecules/m}^{2}\right)$. The specific surface area as determined from the Kr BET data of 7.8448 m^2^ g^−1^ was used in the estimation. Reported values of the surface coverage of LA on flat gold vary from 1.41 × 10^18^ molecules/m^2^ to 2.11 × 10^18^ molecules/m^2^ [43]. In another work from our lab by Allan et al., where LA was self-assembled on a unimodal NPG monolith, the surface coverage of LA on the NPG monolith was calculated from TGA to be 1.31 × 10^18^ molecules/m^2^ [44]. The improved access of LA molecules into hm-NPG is likely to result in greater accumulation of LA inside the material, with molecules chemisorbed onto gold and some physisorbed or trapped, possibly due to the ability of LA to hydrogen bond to itself. It is also possible that the presence of micropores in the material provides more opportunities for LA to become trapped. For the development of electrochemical sensors that require conjugation to proteins, LA is of interest since it has the carboxylic acid functional group and provides a modified electrode surface not as blocked to electron transfer as alkanethiol modification would produce, features we have exploited in some earlier studies [45]. The TGA thermograms of hm-NPG with and without LA loading are shown in Figure 7 (temperature increase of 20 °C/min).

### 3.4. Electrochemical Surface Area Estimation of hm-NPG from Cyclic Voltammetry (CV)

We estimated the electrochemical surface area of hm-NPG using CV by integrating the gold oxide stripping (reduction peak) from the cyclic voltammogram to determine the amount of charge passed. We started by sonicating 0.07 mg hm-NPG in 10 μL water, followed by dropcasting onto the surface of a screen-printed carbon electrode (SPCE). The dropcasted nanoporous material was allowed to dry in ambient conditions. The hm-NPG@SPCE was immersed in 0.5 M H_2_SO_4_, and the CV was scanned between 0 V and 1.3 V, followed by integrating the reduction peak and obtaining a charge of 4.585 mC. specific charge factor in μC/cm^2^ must be applied to convert the charge passed under the gold oxide reduction peak to a surface area. Examining the application of this conversion factor in the nanoporous gold literature, values such as 386 μC/cm^2^, 390 μC/cm^2^, and 450 μC/cm^2^ [46] are found to be used, seemingly with universal agreement. The most prevalent values used recently are 386 μC/cm^2^ or 390 μC/cm^2^. Applying a value of 390 μC/cm^2^, we calculate the electrochemically active surface area to be 1176 mm^2^. In comparison, the geometric area of the active region of the SPC E (d = 4.0 mm) is 12.6 mm^2^. This shows that the ECSA for hm-NPG is almost ten times that of the SPCE alone and would be the dominant contribution for the application of this material in electroanalytical sensor applications. The cyclic voltammogram is shown in Figure S4.

## 4. Conclusions

This study focuses on the fabrication of a hm-NPG monolith with a multimodal pore size distribution (0.58 ± 0.29 μm and 37 ± 10 nm) created using a combination of alloying, annealing, and soft templating techniques. Strong evidence for additional micropores of size 2–3 nm is found from N_2_ adsorption isotherms and the BJH analysis. Such pores are too small to observe with conventional SEM methods, and the sample is too thick for TEM analysis. Given the BET evidence for micropores, it is concluded that the material is trimodal and contains micropores, mesopores, and macropores. Using BET, the prepared monolith’s specific surface area was assessed and was found to be 7.8448 m^2^/g by Kr BET determination. According to TGA, the monolith’s hierarchy increases its loading capacity. Furthermore, our findings confirm how dextran serves well as a framework-generating and reducing agent to create a bicontinuous gold monolith with uniform hierarchical pore morphology throughout a variety of length scales without the need for additional support materials. An open framework template is created when the dextran matrix expands during annealing, and a hierarchical pore structure is created when the dextran matrix is subsequently chemically dealloyed. Materials with hierarchical bicontinuous architecture can be useful in several emerging applications, including energy systems, sensors, and tissue engineering [47]. The hm-NPG material is promising for application in the development of electroanalytical sensors, as it can be used to modify electrode surfaces, providing a high surface area and good electron transfer, and is amenable to being combined with other nanomaterials to create nanocomposite electrodes.

## Data Availability

Datasets are available upon request from the authors.

## Supplementary Materials

The following supporting information can be downloaded at https://www.mdpi.com/article/10.3390/nano16150916/s1. Figure S1: EDS of flat gold before dealloying in nitric acid; Figure S2: EDS of NPG; Figure S3: Pore size measurements of two monolith samples, done to confirm the reproducibility of the fabrication method; Figure S4: Cyclic voltammogram of hm-NPG@SPCE in 0.5 M sulfuric acid; Figure S5: ColorSEM elemental mapping of hm-NPG; Figure S6: hm-NPG in an Eppendorf tube. Table S1: Indexed powder diffraction peaks of Au-Ag alloy; Table S2: Indexed powder diffraction peaks of hm-NPG.
