# Supplementary material for: Facile Fabrication of Hierarchical Multimodal Nanoporous Gold (hm-NPG) via a Polysaccharide Polymer Template Method
Source: Nanomaterials (Basel). 2026 Jul 25;16(15):916. doi: 10.3390/nano16150916 (PMC13468329; doi:10.3390/nano16150916)
Supplement: Supplementary file 1 [file nanomaterials-16-00916-s001.zip › nanomaterials-4418279-supplementary.pdf]

SUPPLEMENTARY INFORMATION

Element	Atomic %	Atomic % Error	Weight %	Weight % Error
Au	19.1	0.5	40.4	1.0
Ag	8.0	0.9	9.3	1.0
Cu	56.2	3.6	38.5	2.5
Zn	16.7	2.1	11.8	1.5

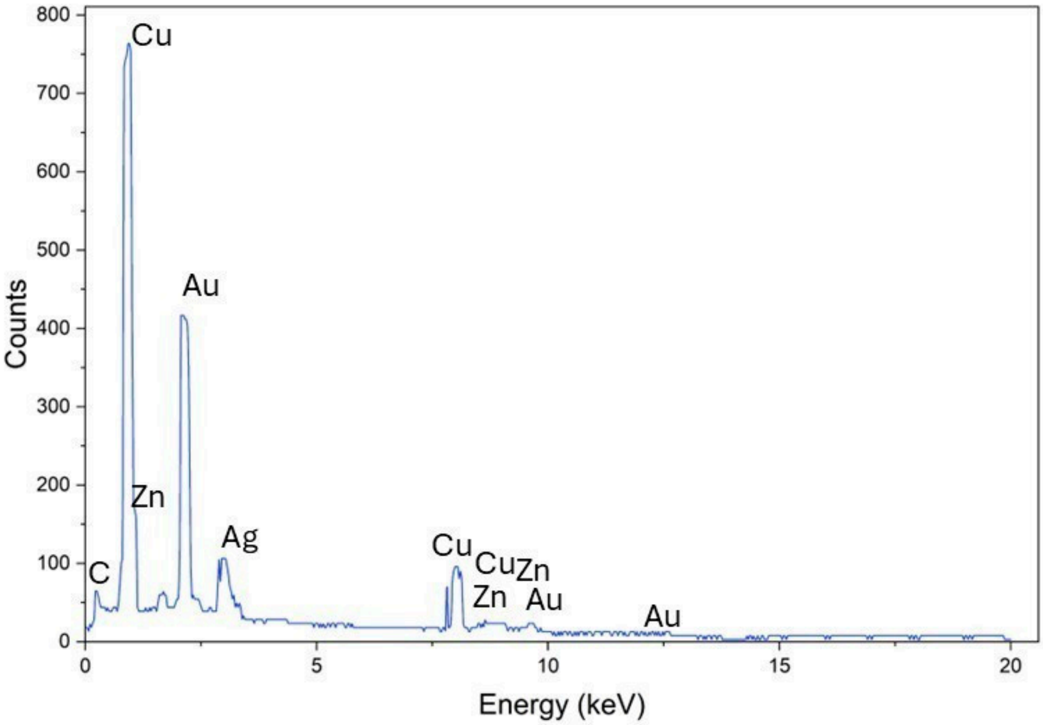

Figure S1. EDS spectrum of 14-carat gold alloy piece before dealloying in nitric acid

Element	Atomic %	Atomic % Error	Weight %	Weight % Error
Au	100.0	1.2	100.0	1.2
Ag	0.0	---	0.0	---

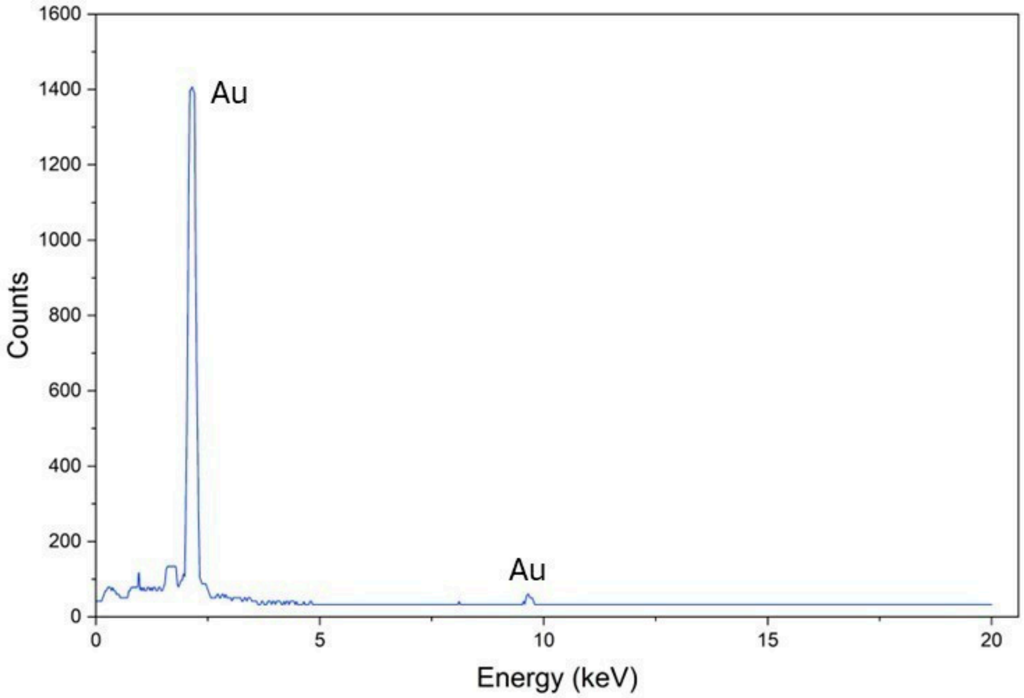

Figure S2. EDS spectrum of NPG after dealloying in nitric acid.

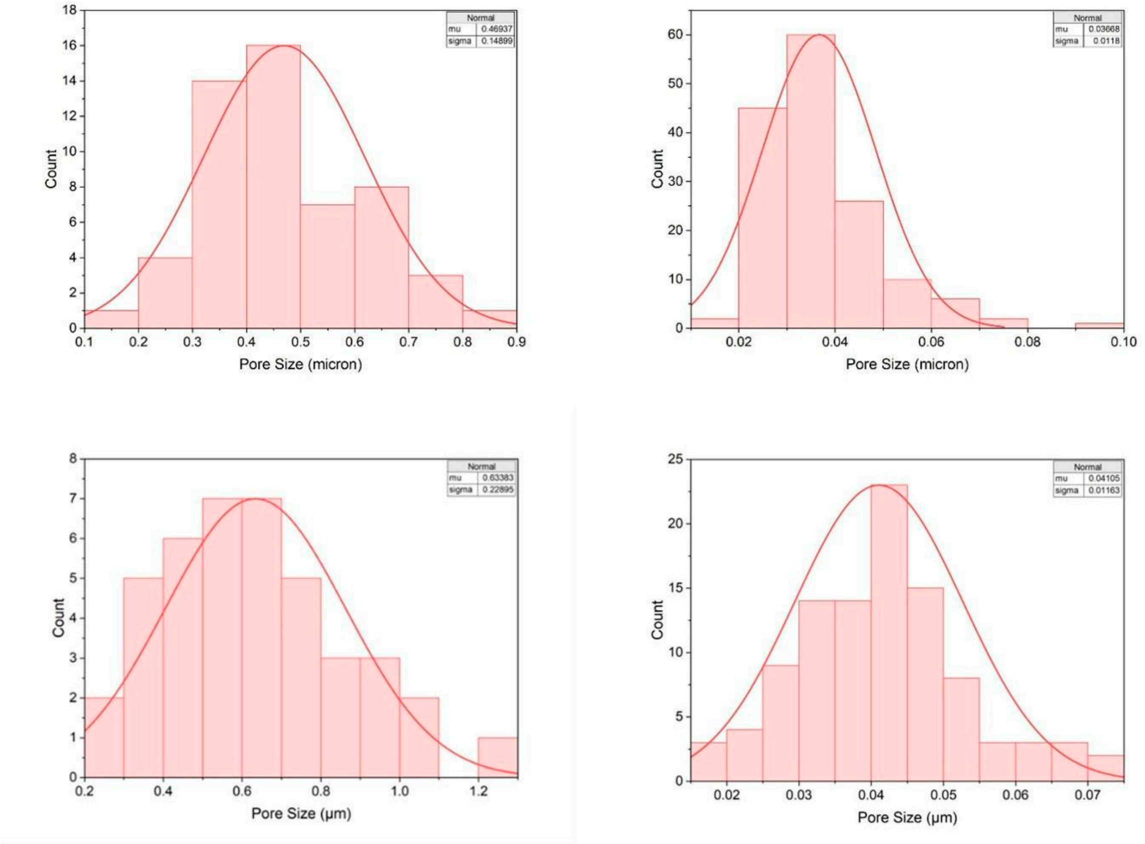

Figure S3. Pore size measurements of two monolith samples, done to confirm the reproducibility of the fabrication method.

Table S1: Indexed powder diffraction peaks of Au-Ag alloy

h k l	2Theta	d (Å)	Height	Area	FWHM (radians)	B (crystallite / grain size) nm	Average (crystallite / grain size) nm
1 1 1	38.071	2.3636	235204	3041537.5	0.01134	13.52	
2 0 0	44.259	2.04643	95225.3	1332561.8	0.01221	12.81	
2 2 0	64.387	1.44691	56323.4	787862.1	0.01221	14.14	13.92(0.8)
3 1 1	77.333	1.23384	60196.7	897047.1	0.01308	14.18	
2 2 2	81.471	1.18132	17583.7	263164.1	0.01308	14.97	

Table S2: Indexed powder diffraction peaks of hm-NPG

h k l	2Theta	d (Å)	Height	Area	FWHM (radians)	B (crystallite / grain size) nm	Average (crystallite / grain size) nm
1 1 1	38.153	2.35686	186928.6	2055924.3	0.00959	15.99	14.35(1.1)
2 0 0	44.348	2.04098	76965.7	1077484.6	0.01221	12.87	
2 2 0	64.542	1.44271	55330	774385.4	0.01221	14.04	
3 1 1	77.529	1.23027	66049.8	987557.6	0.01308	14.21	
2 2 2	81.681	1.17791	20761.8	310247.5	0.01308	14.64	

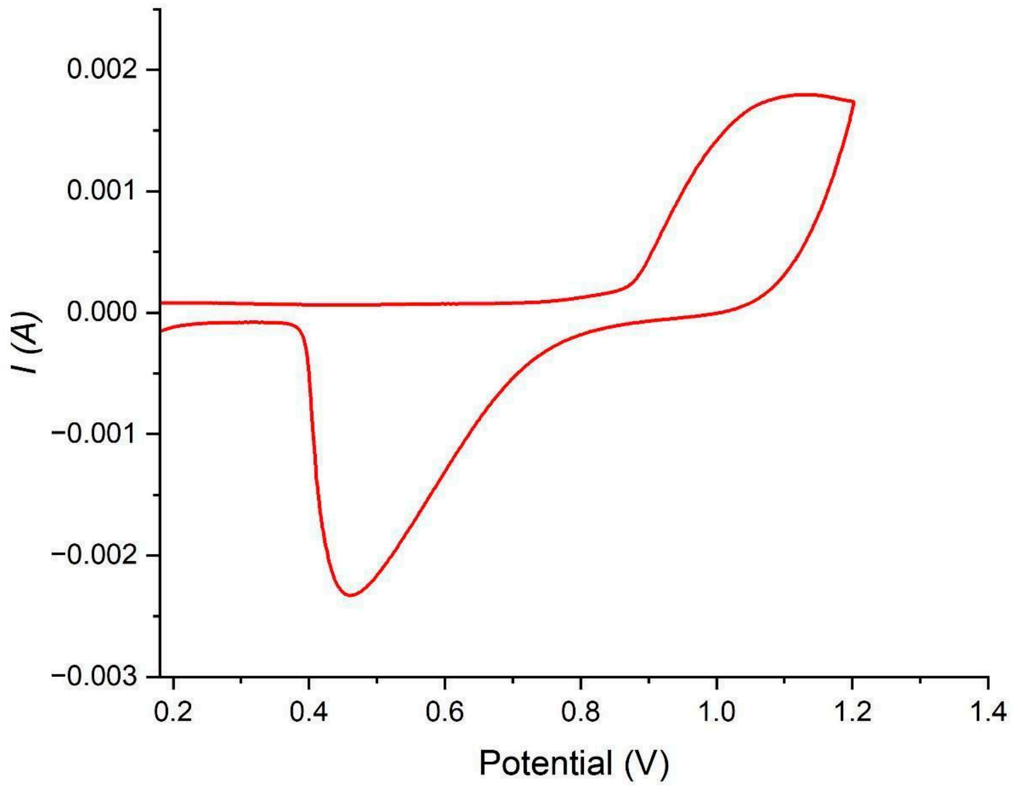

Figure S4. Cyclic Voltammogram of hm-NPG@SPCE in 0.5 M sulfuric acid.

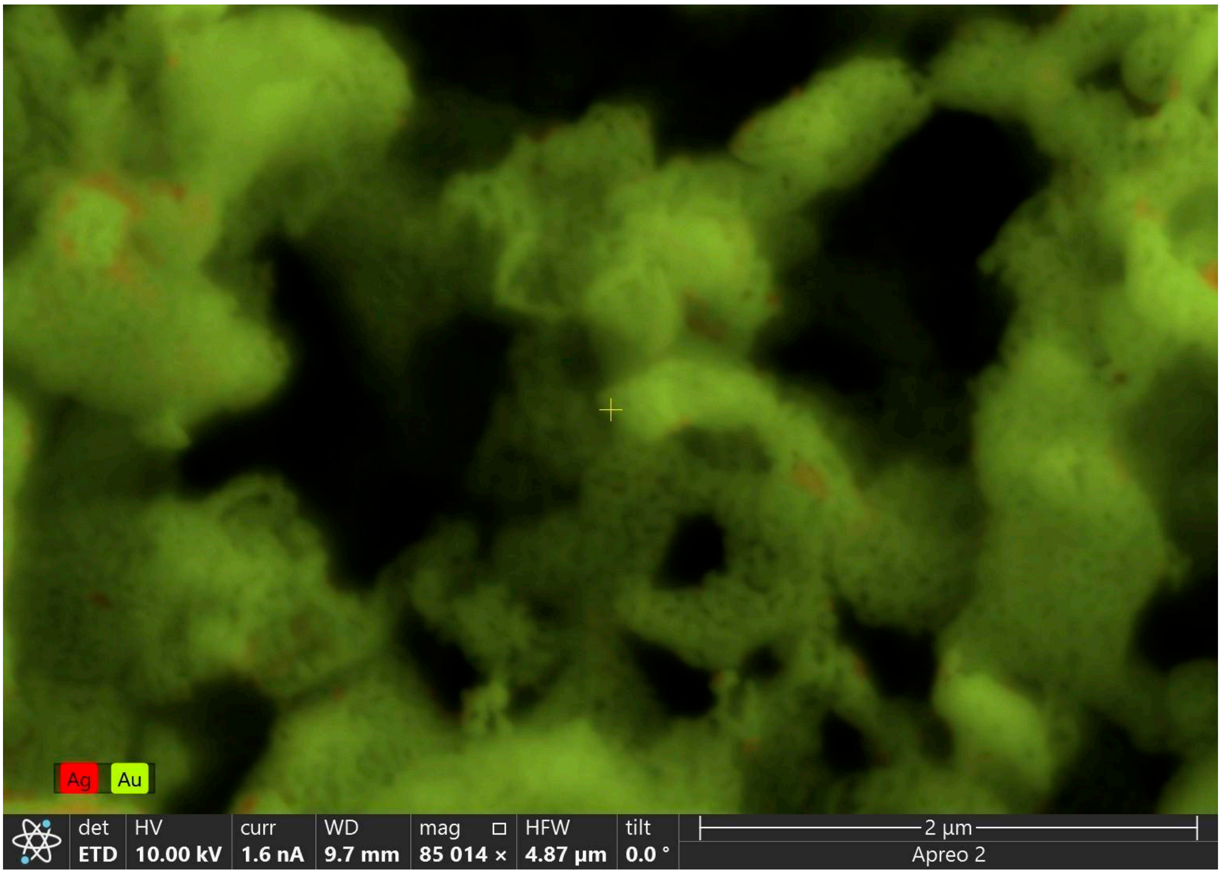

Figure S5. Color SEM elemental mapping of hm-NPG.

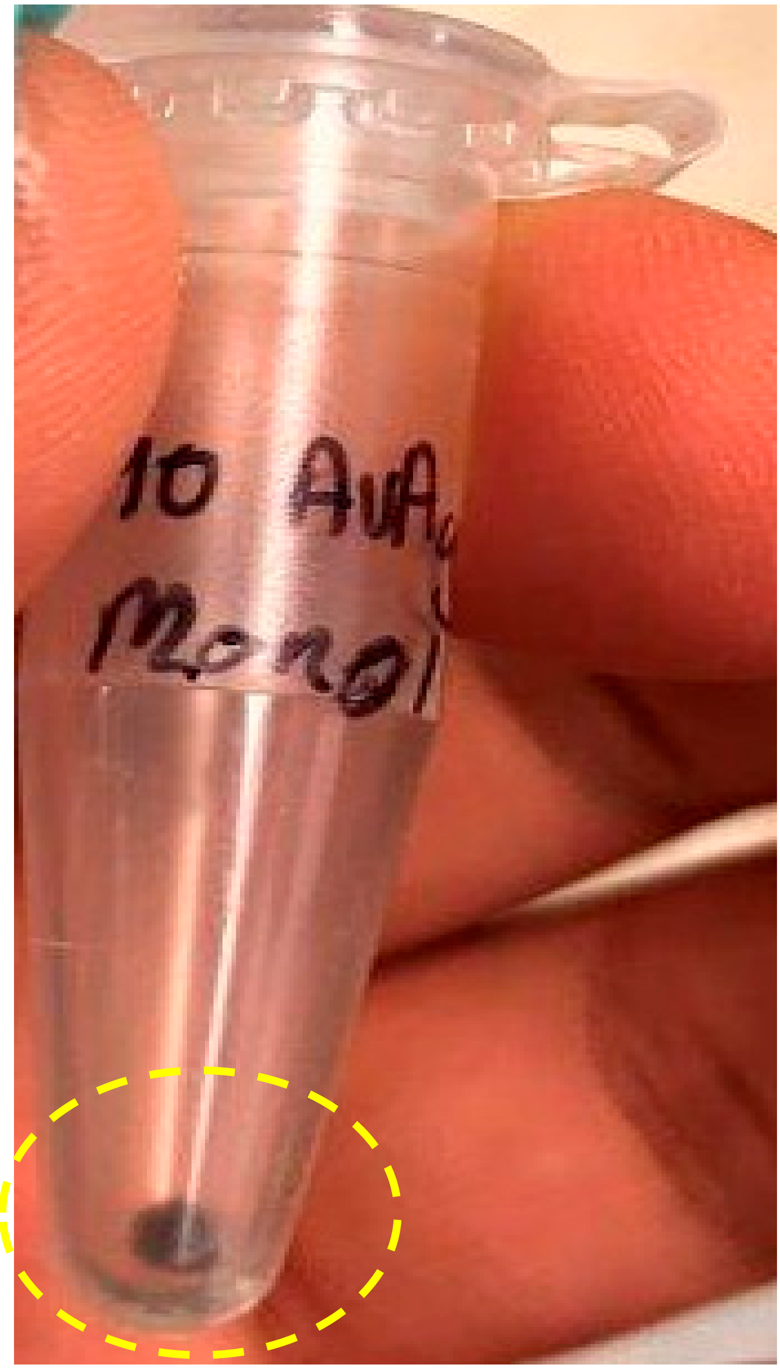

Figure S6. hm-NPG in an Eppendorf tube.
